# Microbial degradation of microplastics: Effectiveness, challenges, and sustainable solutions

**DOI:** 10.1016/j.crmicr.2025.100495

**Published:** 2025-10-18

**Authors:** Priya Yadav, Abhishek Kumar, Kirpa Ram, Ajay Kumar, Rajan Kumar Gupta, Laurent Dufossé

**Affiliations:** a Laboratory of Algal Research, Department of Botany, Institute of Science, Banaras Hindu University, Varanasi 221005, India; b Department of Chemistry, Institute of Science, Banaras Hindu University, Varanasi 221005, India; c Amity Institute of Biotechnology, Amity University, Noida, Uttar Pradesh 201313, India; d Laboratoire CHEMBIOPRO (Chimie et Biotechnologie des Produits Naturels), ESIROI Département Agroalimentaire, Université de la Réunion, 15 Avenue René Cassin—CS 92003, Saint-Denis Cedex 09, 97744 La Réunion, France

**Keywords:** Pollution, Microplastics, Microorganisms, Bacteria, Fungi, Microalgae, Biodegradation

## Abstract

Microplastics (MPs), defined as plastic particles ranging from 1 µm to 5 mm, have emerged as pervasive contaminants in both terrestrial and aquatic environments, posing significant ecological and human health risks. Their sources are broadly categorized as primary, such as microbeads, microfibres, paints and pharmaceutical additives, and secondary, which result from the degradation of larger plastic products. This growing concern of MPs led to development of different mitigation techniques but the recent advancements in microbial biodegradation present a promising approach to mitigating MPs pollution. Thus, microbial biodegradation could play a crucial role in developing sustainable solutions to tackle global MPs pollution. This review explores the potential of various microorganisms, including bacteria, fungi, and algae, in degrading MPs through enzymatic processes, thereby shortening the half-life of these pollutants. A detailed examination of the degradation mechanisms of commonly used polymers such as polyethylene, polystyrene, and polyvinyl chloride highlights the efficacy and limitations of microbial biodegradation. Despite the promising capabilities of certain strains, the overall weight loss rate of MPs remains quite low, typically ranging from 0 to 15%, necessitating further investigation into optimizing enzymatic activity and environmental conditions. Advancements in metagenomics and enzyme engineering offer pathways to enhance degradation efficiency, potentially achieving up to 90% degradation within 10 hrs under optimized conditions. This review underscores the need for comprehensive research to identify key microbial strains and enzymes involved, understand their degradation pathways, and investigate the influence of different environmental matrices on biodegradation processes.

## Highlights

• Bacteria and fungi possess specialized enzymatic systems capable of degrading diverse microplastic polymers, including PET, PE, and PP.
• The efficiency of microbial degradation is influenced by polymer physicochemical properties, environmental conditions, and microbial strains.
• Major limitations include slow degradation kinetics, incomplete mineralization, and accumulation of toxic intermediate compounds.
• Advances in enzyme engineering, microbial consortia optimization, and synthetic biology offer promising avenues to enhance microplastic biodegradation.
• A deeper understanding of microbial ecology, metabolic pathways, and interspecies interactions is essential for microplastic mitigation.

## Graphical abstract

**Figure:**
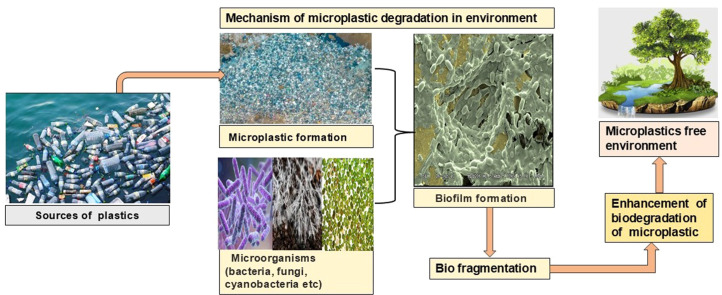

## 1 Introduction

Over the past 150 years, plastics have progressively replaced traditional materials such as wood, glass, and metals, becoming an integral part of modern life. This widespread adoption is primarily due to their exceptional versatility, light weight, and durability (Lebreton and Andrady, 2019 Fayshal, 2024). Unlike metals that are prone to rusting or glass that can easily shatter, plastics offer a more flexible and resilient alternative, making them suitable for an extensive range of applications. These characteristics have made plastics indispensable in numerous industries, including agriculture, where they are used in irrigation systems and packaging; construction, where they serve in insulation and piping; healthcare, where they are critical for medical devices, disposables, and packaging; and consumer goods, where they appear in everything from electronics to clothing (Bundela and Pandey, 2022; Najahi et al., 2025).

The widespread use of plastic materials has resulted in the generation of vast quantities of plastic waste, yet current waste management systems remain insufficient to address this growing challenge (Fayshal, 2024). According to Geyer et al. (2017), by 2015, merely 9 % of global plastic waste had been recycled, 12 % was incinerated, and a staggering 79 % was either landfilled or mismanaged. Ineffective disposal practices have led to the alarming emergence of MPs, plastic particles smaller than 5 mm which are now recognized as significant environmental pollutants. Due to their widespread presence and potential risks to both ecosystems and human health, MPs have attracted growing scientific and public concern (Kumar et al., 2021). These particles are recognized as a major environmental concern due to their pervasive presence and potential harm to ecosystems and human health (Belioka and Achilias, 2024). MPs originate from two primary sources: primary MPs, which are intentionally engineered for use in products such as cosmetic microbeads, textile fibers, and industrial abrasives and secondary MPs, which generated through the gradual degradation of larger plastic debris via physical, chemical, or biological processes (Thompson et al., 2024).

Multiple anthropogenic activities contribute to the growing problem of microplastic (MP) pollution. Major sources include tire abrasion, which generates MPs through friction on road surfaces; laundering of synthetic textiles, which discharges microfibers into wastewater; and inadvertent emissions from industrial processes (Veerasingam et al., 2020). Alarmingly, it is estimated that 80–90 % of MPs present in aquatic environments originate from terrestrial sources, ultimately being transported via runoff and drainage systems into rivers, lakes, and oceans. These microscopic particles, often undetectable without instrumentation, are readily ingested by aquatic organisms, bioaccumulate through trophic levels, and have even been detected in drinking water. Such findings underscore growing concerns regarding their persistent environmental presence and potential adverse effects on both ecological systems and human health (Rakib et al., 2023).

In light of these challenges, addressing plastic pollution, particularly MPs, has emerged as a critical global priority. Effective solutions demand improved waste management practices, innovative in mitigation technologies, and international collaboration to minimize plastic waste at the source. In addition, effective mitigation practices are essential to curb the MPs pollution. Various methods are used to degrade MPs, each with advantages and challenges (Acharya et al., 2022). Thermal degradation involves heating MPs to high temperatures, breaking them down into smaller molecules, though this process requires substantial energy and can release harmful gases (Hu et al., 2022). Hydrolytic degradation uses water to chemically break down MPs, while mechanical degradation relies on physical forces like grinding or milling to reduce plastic size. Both methods are effective but often combined with other techniques to improve efficiency (Sutkar et al., 2023). Advanced Oxidation Processes (AOPs) offer another approach, utilizing highly reactive species such as hydroxyl radicals to degrade MPs. AOP techniques include photodegradation, where ultraviolet (UV) light breaks down MPs, and photocatalytic degradation, which uses catalysts like titanium dioxide (TiO_2_) to accelerate degradation under light exposure (Kim et al., 2022). Exposure to UV radiation can degrade MPs, both biological and non-biological methods have been reported to break down these particles, particularly those measuring up to 5 mm (Sun et al., 2022). Although bioremediation is an effective approach, it presents several limitations. The process is often time-consuming, applicable primarily to biodegradable compounds (Alqahtani et al., 2023; Le et al., 2023). Moreover, scaling up biological treatment systems poses significant challenges. However, integrating biodegradation with other remediation methods has shown promising results (Kumah et al., 2023; Shruti et al., 2023; Tse et al., 2023).

Biodegradation involves the use of microorganisms such as microalgae, bacteria, and fungi to break down MPs into less harmful substances. Approaches like bioaugmentation, which introduces specialized microbial strains into contaminated environments, have shown promise in enhancing the natural degradation process (Tang, 2023). Composting, another biodegradation strategy, involves the use of microbial communities in organic waste decomposition, promoting the breakdown of MPs alongside compost material. Microbial strains like *Acinetobacter* sp., *Bacillus* sp., and *Pseudomonas* sp. have exhibited significant capabilities in degrading various types of plastics, including polypropylene (PP), polyethylene (PE), and polyethylene terephthalate (PET) (Wróbel et al., 2024). Therefore, this review includes an overview of these combined approaches. Microorganisms like microalgae, bacteria, and fungi are acknowledged as economically feasible and environmentally friendly agents for the bio-deterioration of MPs. This review provides a comprehensive assessment of microplastic degradation processes. It outlines the definition, classification, sources, toxicity, physicochemical properties, and environmental persistence of microplastics to establish a foundational understanding of the problem. Additionally, paper addresses the key limitations and challenges, including environmental factors, scalability concerns, and the risk of secondary pollutants. The review further discusses emerging approaches and future perspectives, emphasizing advanced technologies, integrated strategies, and sustainable solutions aimed at enhancing degradation efficiency and mitigating long-term ecological impacts.

## 2 Definition and type of MPs

The term "microplastics" was first introduced in the 1990 by an African researcher in the seminal paper "Plastic and other artifacts on South African beaches: temporal trends in abundance and composition." Since then, the term has achieved widespread global usage, broadly referring to small plastic particles. Although debates persist regarding a standardized definition, MPs are generally described as plastic fragments ranging from 1 µm to 5000 µm in size (Jaafar et al., 2020; Khalid et al., 2021). Based on size classification, plastic debris larger than 25 mm is categorized as macroplastics, particles between 5–25 mm as mesoplastics, and those smaller than 100 nm as nanoplastics (Ziani et al., 2023). MPs are further classified into two major types based on their origin: primary and secondary MPs. Primary MPs are deliberately manufactured at small sizes for use in personal care products (e.g., toothpaste, facial cleansers, and shower gels) and industrial applications such as abrasive agents in air-blasting processes and resin pellet production (Khalid et al., 2021; Nava and Leoni, 2021). In contrast, secondary MPs are generated through the fragmentation of larger plastic items due to various degradation pathways, including chemical processes (e.g., UV exposure, freeze-thaw cycles), physical forces (e.g., wave action, turbulence), and biological mechanisms (e.g., microbial degradation) (Khalid et al., 2021; Dong et al., 2021). The origin of all MPs ultimately traces back to the polymerization of monomeric units during plastic production.

## 3 Sources of MPs in the environment

**Fig. 1:**
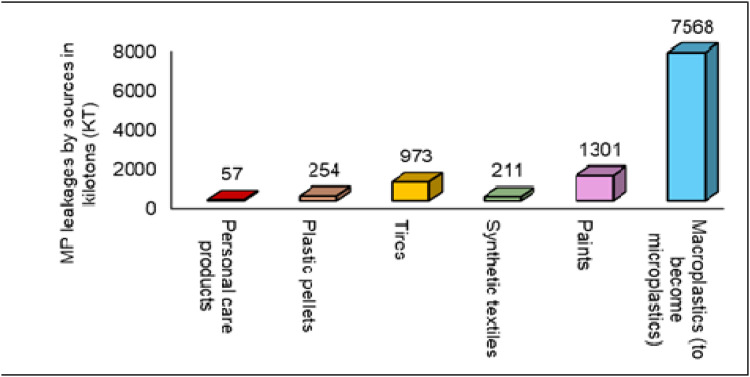
Contribution of MPs from different sources to the marine environment annually.

MPs contamination stems from both primary and secondary sources, representing one of the most pressing environmental challenges of our time. Primary sources include cosmetic products, household goods, and drug delivery systems containing polymerized materials such as polyethylene (PE), polyamide nylon (PN), polystyrene (PS), polyvinyl chloride (PVC), and polypropylene (PP) (Rani, 2022). Within personal care items, MPs typically sized between 0.1 and 0.5 mm are prevalent as "microbeads" or "micro-exfoliants" (Bermúdez and Swarzenski, 2021; Cozzarini et al., 2023). These materials require antioxidants and stabilizers to prevent oxidative-thermal deterioration. Secondary MP formation occurs through physical wear, hydrolysis, and biodegradation, with organisms facilitating decomposition through enzyme production (Yang et al., 2024). Notable contributors to the dissemination of secondary micro and nano-plastics include deliberate disposal of large polymer items into terrestrial and aquatic ecosystems, as well as industrial processes such as the thermal degradation of polystyrene and synthetic fibers (Dimassi et al., 2022; Saadu et al., 2023). The widespread proliferation of disposable plastic products, particularly those constructed from PE, polyester fibers, and PET, has resulted in excessive production levels of these materials. According to Thompson et al. (2024), microplastics enter marine environments from multiple sources totaling 0.8–3 million tons annually (Fig. 1). The largest contributors include synthetic textiles from washing machines (200,000–500,000 tons/year, ∼35 % of primary microplastics), tire wear particles (270,000–1,300,000 tons/year), and fragmentation of larger plastic debris (7.6 million tons of MPs input that eventually degrades). Smaller but significant sources include paint particles (15,000–100,000 tons/year), personal care products (8000–40,000 tons/year), and fishing gear abrasion. Land-based sources account for approximately 80 % of total marine plastic pollution, with sea-based activities contributing the remaining 20 % (Thompson et al., 2024).

Each year, approximately 1–2 million metric tons of MPs are introduced into the marine environment, posing a critical threat to oceanic ecosystems. The primary contributors include synthetic textile fibers (∼35 %), tire and road wear particles (∼28 %), and discarded or lost fishing gear (∼10 %) (Yang et al., 2021). Other significant sources encompass microbeads from personal care products, plastic pellet spills, and degraded paint particles. Collectively, these inputs underscore the pervasive and multifaceted nature of MPs pollution in marine systems.

### 3.1 Household and agricultural sources

Household waste encompasses diverse disposable materials including shopping bags, food packaging, printed materials, diapers, and organic waste. This category distinguishes between non-hazardous materials (food waste, paper, bottles) and hazardous waste (plastics, electronics, medical waste) containing toxic organic compounds, heavy metals, and ozone-depleting substances that pose flammability and explosive risks, necessitating specialized disposal procedures (Dehghani et al., 2021; Kumar et al., 2023). Hazardous waste often contains toxic organic compounds, high levels of heavy metals, and ozone-depleting substances, posing potential risks of flammability or explosiveness and necessitating specialized collection, handling, and disposal procedures to minimize environmental contamination and protect public health (Chauhan et al., 2024). Effective management of both hazardous and non-hazardous household waste is therefore critical to ensure environmental sustainability and reduce associated health risks.

Plastics have become essential in agricultural practices, serving various purposes such as irrigation, mulching, crop protection, silage covering, and packaging for seeds and fertilizers. Additionally, a notable portion of plastic is utilized in creating nets and coverings to safeguard crops from adverse weather conditions, wildlife, and insects (Espí et al., 2006). The global yearly consumption of agricultural plastics stands at approximately 6.5 million metric tons, resulting in a significant volume of agricultural plastic waste. While this accumulation presents a challenge in terms of pollution, it also offers an opportunity for consolidation and processing (Lakhiar et al., 2024). Nevertheless, the recycling of agricultural plastic waste remains minimal, with recycling rates varying greatly between countries and contingent upon regional facility availability. A considerable portion of agricultural plastic waste is disposed of through burial in soil, uncontrolled burning in fields, or abandonment in open areas, leading to its eventual accumulation in landfills or adjacent water bodies such as rivers and canals (Lanorte et al., 2017; Islam et al., 2023).

### 3.2 Road dust and industrial contributions

Road dust is recognized as a major source of MPs, defined as plastic particles smaller than 5 mm (Myszka et al., 2023). The presence of MPs in road dust raises environmental and health concerns, as they can be transported to aquatic ecosystems, contributing to widespread pollution. These MPs primarily come from various human activities. Vehicle tires release rubber and other materials through wear, significantly contributing to the MPs load, with studies finding between 102 and 303 particles per gram of road dust. Road materials, such as bitumen in asphalt and road paint, also contribute, with carbon black a common additive in tires and bitumen identified as a key component of MPs in road dust (Giechaskiel et al., 2024; Özen and Mutuk, 2025). Additionally, plastic litter on roads breaks down into smaller fragments, further increasing MPs content. Research shows that MP concentrations in road dust vary with environmental conditions (Premarathna et al., 2025). For instance, longer drying periods after rainfall lead to higher concentrations, reaching up to 1530 MPs per gram over three days. Land use also impacts MP levels, with industrial areas showing concentrations as high as 2410 particles per kilogram (Yang et al., 2023; Yamamoto et al., 2025). Most MPs in road dust are small, often less than 400 µm, posing health risks due to inhalation (Kang et al., 2022; Tejano et al., 2025). MPs are transported through stormwater runoff, which carries them from roads to aquatic systems, and environmental factors like traffic volume, temperature, and wind influence their distribution.

Industrial plastic waste originates from large-scale manufacturing, processing, and packaging industries, including construction and demolition firms, electronics and electrical sectors, automotive manufacturers, packaging companies, as well as small and medium enterprises. The benefit of plastic waste from these sectors is its typically clean and uncontaminated nature and its availability in significant quantities. However, effective guidelines and regulations are imperative for the proper disposal and recycling of industrial plastics to ensure optimal utilization with minimal environmental impact (Fayshal, 2024; Kibria et al., 2023). Municipal solid waste (MSW) is characterized by its heterogeneous composition, comprising a mixture of recyclable, biodegradable, and hazardous materials. Reprocessing and reshaping represent efficient strategies for managing homogeneous plastic waste, as opposed to disposing of or incinerating it alongside general MSW (Gaylor et al., 2013). Conversely, heterogeneous plastic waste, containing mixed resins, presents challenges for reclamation due to the varying processing temperatures and pressures required for each resin type.

### 3.3 Medical and personal care products

Medical plastic waste (MPW) has increased substantially due to technological advancement and healthcare expansion, encompassing PPE, gloves, syringes, catheters, and medicine coverings (Thacharodi et al., 2024). In 2018, China generated approximately 817,000 tons of MPW (Ding et al., 2021), while the COVID-19 pandemic exacerbated this issue dramatically. Hubei Province saw 370 % increases during outbreak peaks (Ding et al., 2025; Klemeš et al., 2020). The COVID-19 pandemic dramatically intensified this issue. For instance, UNICEF distributed over 200 million medical masks globally beginning in January 2020, significantly adding to the MPW burden (Filip et al., 2022). The discovery of various types of facemasks in the ocean, even within bird nests, has tragically resulted in the deaths of some birds due to entanglement in this debris. Amid the COVID-19 pandemic, the United States witnessed a substantial increase in medical mask usage, reaching 89 million, while the United Kingdom utilized nearly 24.37 billion masks annually (Wang et al., 2023). The growing volume of plastic waste poses a significant threat in terms of carbon dioxide emissions. China and Japan collectively employ approximately 14.8 million facemasks daily (Dey et al., 2023). The disposal of facemasks raises alarming biodiversity concerns, potentially harming the digestive systems of animals and causing internal blockages. The release of micro-particles from mask usage has surged to 1246.62 items, emphasizing the urgency of action (Chen et al., 2022). Single-use plastics including facemasks may take over 450 years to decompose, releasing MPs during degradation and contributing to an estimated 1.56 billion masks entering oceans (Benson et al., 2021; Jiang et al., 2023; Silva et al., 2020;). During the COVID-19 crisis, there was an increase in plastic burning to prevent contamination, resulting in adverse environmental effects (Filip et al., 2022; Jayasinghe et al., 2024).

PCPs play a major role in MPs pollution, especially due to the presence of microbeads. These tiny plastic particles are deliberately included for their exfoliating or cleansing benefits (Sun et al., 2020). Commonly used polymers for these microbeads include PE, PP, PET, polymethyl methacrylate, various nylons, polyester, and polyurethanes (Bharath et al., 2021). MPs can be found in a variety of personal care items such as facial cleansers, body washes, shampoos, conditioners, toothpaste, and cosmetics like makeup and sunscreens (Bikiaris et al., 2024). Research in Sri Lanka identified low-density polyethylene and ethylene-propylene copolymer as prevalent types of MPs, with sizes ranging from 150 to 600 μm (Gamage and Mahagamage, 2024). In India, microbeads were present in 45 % of products tested, with polyethylene being the most frequently used polymer (Sun et al., 2020). Additionally, oral care products exhibited considerable MPs contamination, with toothbrushes showing the highest concentration of particles (Protyusha et al., 2024).

## 4 MPs pollution and need for its mitigation

MPs have emerged as a pressing environmental concern, particularly within marine ecosystems. Their pervasive presence in oceans, rivers, and other aquatic environments stems from multiple sources, including the fragmentation of macroplastics, the use of microbeads in personal care products, and the release of synthetic fibers during textile laundering (Wu et al., 2018). Owing to their minute size, MPs are readily ingested by a wide range of marine organisms ranging from plankton at the base of the food web to larger fauna such as fish, seabirds, and turtles posing serious risks to biodiversity and food security (Grattagliano et al., 2025). This ingestion can severely affect their health and survival. One major concern is the blockage of gastrointestinal tracts caused by MPs, which leads to decreased food intake and starvation, particularly in fish and seabirds (Fournier et al., 2021). This obstruction disrupts normal feeding behaviors and creates energy imbalances that hinder reproduction and growth. Research indicates that exposure to MPs can result in stunted growth and increased oxidative stress in marine organisms, damaging vital cellular functions and weakening immune systems (Bessa et al., 2018; Bharath et al., 2021; Koelmans et al., 2016).

**Fig. 2:**
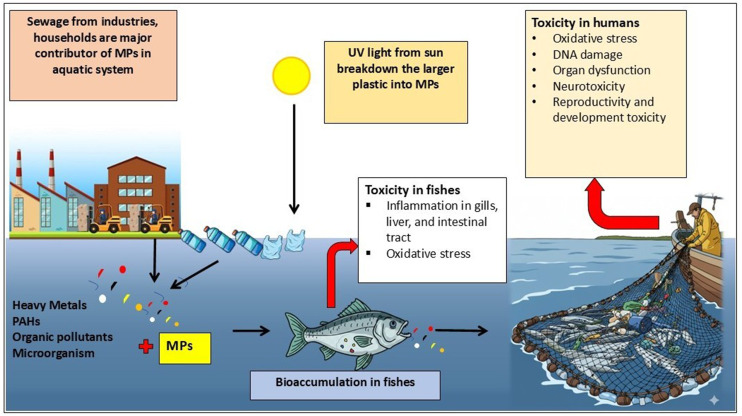
Risk of MPs and its associated pollutants in different organism of aquatic system.

MPs also act as vectors for toxic chemicals by absorbing persistent organic pollutants (POPs) and heavy metals from the environment. When ingested, these toxins can leach into marine organisms' tissues, leading to biochemical disruptions such as endocrine interference and reproductive issues (Wang et al., 2018). Bioaccumulation occurs when toxins concentrate within an organism's tissues, while biomagnification amplifies these effects as MPs move up the food chain, impacting larger predators and potentially humans who consume seafood (Miller et al., 2020). Humans can be exposed to MPs through ingestion, inhalation, or skin contact, leading to various health issues including inflammation and oxidative stress (Castro-Castellon et al., 2022). The environmental impact of MPs is extensive; they disrupt nutrient cycling and introduce harmful pollutants into food webs. Addressing MPs pollution is crucial for protecting ecosystems, marine life, and human health (Grattagliano et al., 2025). Additionally, it has economic implications for industries like fishing and tourism. Global regulations are increasingly advocating for sustainable practices to mitigate plastic waste, aligning with broader sustainability efforts (Mitrano and Wohlleben, 2020). Fig. 2. Shows MPs and their associated pollutants accumulate across aquatic organisms, causing physical damage and serving as carriers of toxic substances, which lead to bioaccumulation, trophic transfer, and potential ecological and human health risks (Amelia et al., 2021).

### 4.1 Mechanism of MPs degradation in environment

MPs biodegradation is a complex process influenced by both physicochemical and microbiological factors across diverse environments. The biodegradation of MPs involves three primary phases: (a) the initial attachment of microorganisms onto the MPs surface, (b) the utilization of the MPs as a carbon source, and (c) the subsequent breakdown of the MPs itself (Lucas et al., 2008). Upon entering aquatic environments, MPs interact with inorganic particles, microbes, and organic matter, providing surfaces for microbial colonization and biofilm formation, thus creating new ecological niches (Sooriyakumar et al., 2022).

**Fig. 3:**
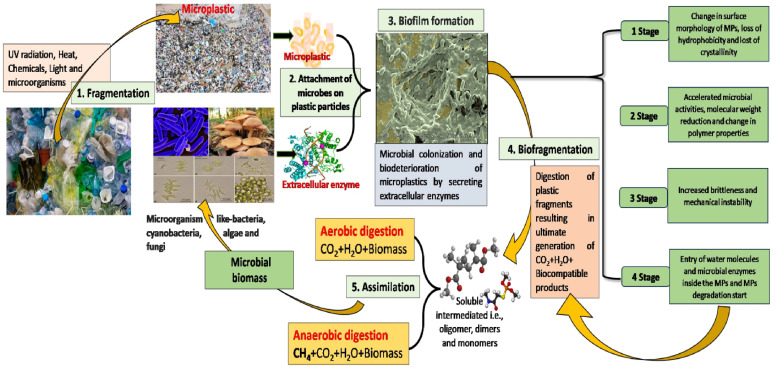
Illustration of general mechanism of microplastics degradation by microorganisms.

In the first stage, microorganisms attach themselves to MPs surfaces, causing changes in their surface properties like adhesion and hydrophilicity (Fig. 3). A diverse array of microorganisms such as fungi, protists, bacteria, and algae, can attach to MPs surfaces and leading to formation of biofilm (Tsiota et al., 2018; Sooriyakumar et al., 2022). Biofilm formation is a key mechanism in MPs biodegradation as it facilitates the attachment of microorganisms to MP surface, promotes the production of degradative enzymes and generates organic by products (Zurier and Goddard, 2021). This is followed by the release of chemicals and monomers from the MPs due to microbial enzyme activity and cellular processes, which accelerates the degradation process in the second stage. This enzymatic breakdown results in the conversion of large polymers into smaller monomers and oligomers, which have reduced molecular weights (Mohanan et al., 2020). Eventually, microbial filaments and water infiltrate the MPs, facilitating their decomposition and utilization by microorganisms. Through these stages, microorganisms adhere to the surfaces of polymers and enzymatically degrade them, obtaining energy for their growth in the process (Elahi et al., 2021). This comprehensive interaction and breakdown mechanism underscore the significant role that microorganisms play in the biodegradation of MPs in various environments.

## 5 Microorganisms in MPs degradation

Microorganisms with the ability to break down MPs have been identified in a variety of environments, such as soil, freshwater, and marine ecosystems, illustrating their potential for MPs biodegradation (Kothawale et al., 2023). These microorganisms, which includes algae, fungi, and bacteria, utilize specific metabolic pathways to degrade MPs (Jain et al., 2023; Yuan et al., 2020). A critical component of this degradation process involves enzymes that these microorganisms produce. For instance, certain bacterial species release extracellular enzymes capable of hydrolyzing plastic polymers into smaller, more biodegradable components (Heris, 2024). Mishra et al. (2022), have provided insight into the widespread occurrence of these microorganisms in different ecological niches, highlighting their importance in tackling plastic pollution. Algae, for example, have been found to secrete oxidative enzymes that initiate the breakdown of synthetic polymers. Fungi also play a crucial role by secreting ligninolytic enzymes, which can degrade various plastic types (Vingiani et al., 2019). Similarly, bacterial species are known to produce hydrolases, such as esterases and lipases, which target the bonds in plastic molecules, accelerating their breakdown into simpler monomers (Cai et al., 2023). Choi et al. (2024), has emphasized isolating these enzymes to explore their efficiency and to understand the molecular mechanisms of MPs degradation. The following sections provide a detailed explanation of the biodegradation pathways for MPs by bacteria, fungi, algae, and other microorganisms.

### 5.1 Bacteria

**Fig. 4:**
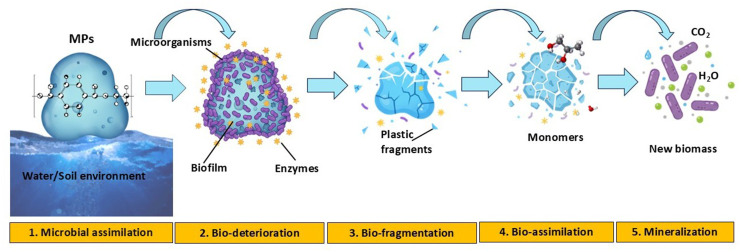
Degradation of MPs using bacterial enzymes including steps like (1) Microbial assimilation, (2) Bio-deterioration, (3) Bio-fragmentation, (4) Bio-assimilation, and (5) Mineralization.

Bacteria are the predominant microorganisms found in soil, air, and water, known for their adeptness in breaking down pollutants. Bacterial strains are proficient in degrading MPs and have been unearthed across diverse habitats, spanning polluted soil, marine sediments, wastewater, compost, sludge, municipal landfills, mangroves, and regions characterized by extreme climatic conditions (Awasthi et al., 2020; Rajendran et al., 2022). Studies have identified Sporosarcina globispora and Bacillus cereus isolates from mangrove sediments as promising candidates for PP degradation (Helen et al., 2017). Similarly, Auta et al. (2017) highlighted the MPs-degrading capabilities of Bacillus gottheilii and B. cereus across various MP polymers. B. cereus demonstrated weight reductions of 1.6 % for PE, 6.6 % for polyethylene terephthalate (PET), and 7.4 % for polystyrene, while B. gottheilii exhibited reductions of 5.8 % (PS), 6.2 % (PE), 3.6 % (PP), and 3.0 % (PET). More recently, Bacillus sp. Y-01, isolated from plastic-contaminated sites in the Yellow Sea, demonstrated the ability to utilize PP as its sole carbon source (Arefian et al., 2020). In another study, Patil and Bagde (2012) observed partial mineralization of PVC to the extent of 8.87 % by *Micrococcus* sp. Similarly, Giacomucci et al. (2019) demonstrated the PVC-degrading ability of *Pseudomonas citronellolis* and *Bacillus fexus*. However, their findings suggested that the biodegradation activity primarily targeted additives within the PVC MPs, with less emphasis on the polymer chains themselves. In a notable study, a marine bacterial strain, *Alcanivorax borkumensis*, characterized by hydrocarbonoclastic properties, was employed to demonstrate biofilm formation on films of low-density polyethylene (Davoodi et al., 2023). The biodegradation process was enhanced by the presence of pyruvate, yeast extract, and hexadecane, which facilitated microbial activity. The interaction of alkanes with plastic surfaces modifies the hydrophilicity of microbial cell membranes, promoting the introduction of functional groups such as carboxyl (–COOH), hydroxyl (–OH), and carbonyl (C=O) (Miho et al., 2018). These chemical modifications increase surface reactivity and significantly accelerate the biodegradation of polyethylene (Miho et al., 2018). Fig. 4 illustrates the sequential process of MPs degradation mediated by bacterial enzymes. The pathway begins with microbial assimilation, where bacteria colonize the plastic surface. This is followed by bio-deterioration, involving structural changes and weakening of the polymer surface. Subsequently, bio-fragmentation breaks down large polymers into smaller oligomers and monomers. These intermediates undergo bio-assimilation, where microbial cells take them up and metabolize them. Finally, the process culminates in mineralization, leading to the conversion of MPs into inorganic compounds such as CO₂, H₂O, and biomass (Mandal et al., 2024). Notably, Bacillus spp. in combination with Pseudomonas spp. represent approximately 21 % of the bacterial taxa implicated in MPs degradation.

Exposure to *Stenotrophomonas maltophilia* LB 2–3 led to rapid molecular weight reduction and physical property alterations in polylactic acid (Jeon and Kim, 2013). MPs like polyurethanes when cultured with *Escherichia coli*, exhibited degradation rates ranging from 1 % to 2 % after 72 h of incubation (Habib et al., 2020; Jeyavani et al., 2021). Additionally, *Microbacterium paraoxydans* and *Pseudomonas aeruginosa* have exhibited an effective biodegradation of low-density polyethylene over a two-month period. Biofilms generated by AKS2 *Pseudomonas* sp. have demonstrated the ability to degrade PE by up to 5 % within forty-five days without prior treatment (Montazer et al., 2020). Another notable strain, C208 *Rhodococcus ruber*, has shown a biodegradation rate of 0.86 % per week. Furthermore, bacterial consortia such as *Bacillus* sp. and *Paenibacillus* sp. have achieved a reduction in the dry weight of MPs by up to 15 % within sixty days (Montazer et al., 2020). When evaluating the MPs degrading efficacy of *Streptomyces* sp. and *Arthrobacter* sp. strains isolated from agricultural soil, researchers found that the microbial consortium outperformed treatments with individual strains (da Silva et al., 2024). Numerous studies have documented the biodegradation of polypropylene by microbial strains such as *Bacillus subtilis, Rhodococcus rhodochrous, Pseudomonas stutzeri, Bacillus fexus,* and other isolates such as *Achromobacter xylosoxidans* which form biofilms (Sutkar and Dhulap, 2025). For example, a study demonstrated that Rhodococcus and Bacillus strains isolated from mangrove sediments achieved PE degradation efficiencies of 4.0 % and 6.4 %, respectively, after 40 days of incubation (Cai et al., 2023; Han et al., 2020). Moreover, *Pseudomonas, Lysinibacillus fusiformis,* and *Chelatococcus* have been linked to polypropylene degradation across diverse ecological settings (Cai et al., 2023).

Bacteria play a crucial role in bioremediation due to their diverse metabolic pathways, especially in the context of MPs degradation, as highlighted in this review. Among them, biofilm-forming bacteria like *Rhodococcus ruber* show promise for plastic degradation. *Rhodococcus ruber* produces laccase enzymes that aid in depolymerizing polyethylene by oxidizing its polymer backbone. Over an eight-week period, these bacteria developed biofilms on polyethylene surfaces and degraded approximately 7.5 % of the polymer (Skariyachan et al., 2018). In a study conducted by Muhonja et al. (2018), low-density polyethylene (LDPE) sheets with thicknesses of 30 and 40 µm were exposed to pure bacterial cultures isolated from a dumpsite. The isolates belonged to several genera, including Cellulosimicrobium, Ochrobactrum, Bacillus, Lysinibacillus, Pseudomonas, and Brevibacillus. Following 16 weeks of incubation, Brevibacillus borstelensis and B. cereus demonstrated the highest LDPE degradation efficiencies, achieving weight loss rates of 35.7 % and 20.4 %, respectively (Du et al., 2024).

Generally, thinner sheets of LDPE demonstrated higher degradation rates, suggesting that sheet thickness significantly influences degradation rates. Kyaw et al. (2012) investigated the degradation of LDPE films using four bacterial strains: *Pseudomonas syringae, Pseudomonas putida*, and *Pseudomonas aeruginosa*. After 120 days of incubation, all tested bacteria facilitated degradation, with *P. aeruginosa* exhibiting the highest weight loss of 20.0 %. The degradation of PE by *Pseudomonas* strains was attributed to the enzymatic activities of alkane hydroxylase and reductase, pivotal in polyethylene depolymerization (Jeon and Kim, 2015). Furthermore, Rajandas et al. (2012) documented even more substantial degradation of LDPE by *Microbacterium paraoxydans* and *P. aeruginosa*.

For instance, Skariyachan et al. (2021) showcased the biodegradation of PE by a bacterial consortium comprising *Pseudomonas* and *Enterobacter*. Auta et al. (2018) identified biofilm-forming bacteria such as *Bacillus* sp., and *Rhodococcus* sp. which exhibited the capability to degrade PP. Following a 40-day incubation period, PP microparticles treated with *Bacillus* sp. and *Rhodococcus* sp. experienced weight losses of 6.4 % and 4.0 %, respectively, alongside the emergence of pores and irregularities on the PP surface. Aravinthan et al. (2016) conducted a study utilizing polypropylene particles treated with thermal and UV-irradiation. In their study, Aravinthan et al. (2016) employed two bacterial consortia *Pseudomonas azotoformans* with *Bacillus flexus*, and *Bacillus subtilis* with *Bacillus flexus* to evaluate PP degradation over a 12-month exposure period. Although the study did not assess the biodegradation of untreated PP, notable degradation was observed in pretreated PP samples, with UV irradiation pretreatment yielding the most pronounced effects. The authors proposed that microbial degradation of polypropylene likely occurred through the oxidation of short-chain polymer fragments, facilitated by the pretreatment process.

Other key genera involved include Exiguobacterium sp., Pseudomonas chlororaphis, and Ideonella sakaiensis (Adithama et al., 2023). It is evident that bacteria sourced from diverse environments such as contaminated soil, microbiota, municipal landfills, wastewater, sewage, compost, and extreme habitats have demonstrated the ability to degrade MPs (Martak et al., 2024). Both microbial consortiums and pure cultures have been employed in investigating bacterial-assisted MPs breakdown, with bacterial consortia being particularly noteworthy for their enhanced efficacy and community stability.

### 5.2 Fungi

The fungal kingdom encompasses a vast and diverse group of organisms, primarily consisting of saprotrophs, opportunistic parasites, and obligate parasites. Their exceptional adaptability allows them to colonize a broad range of habitats, from aquatic to terrestrial ecosystems, and to persist under various climatic conditions (Zhai et al., 2023). Fungi are capable of producing a wide spectrum of organic biosurfactants and extracellular enzymes including hydrophobins, which play a crucial role in the degradation of complex polymers into simpler monomers (Dinakarkumar et al., 2024). This enzymatic activity not only enables fungi to access essential carbon and electron sources but also contributes to the breakdown and mineralization of persistent environmental contaminants. Additionally, fungi exhibit notable resistance to toxic chemicals and heavy metals, further underscoring their potential in bioremediation applications (Srikanth et al., 2022).

**Fig. 5:**
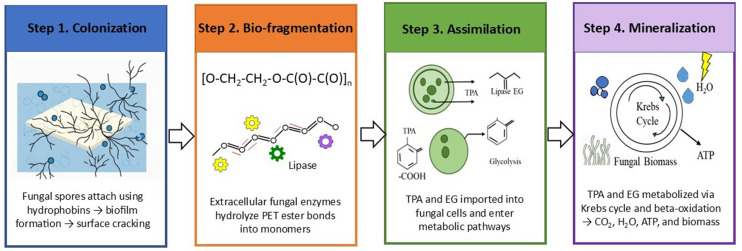
Illustrates the comprehensive mechanism of fungal plastic biodegradation.

Several fungal isolates from an estuary displayed active MP degradation capabilities. Proponents of bacteria highlight their rapid growth rates and superior adaptation to toxic conditions, suggesting they are more efficient degraders. Fungi can grow on a wide variety of substrates and are well-suited to the "plastisphere" due to their absorptive mode of nutrition. Their apical growth and ability to form biofilms enhance their degradation capabilities. Fungal cells secrete hydrophobic proteins, enabling them to utilize polymers in MPs as sources of carbon and energy (Kasuya et al., 2009; Olicón-Hernández et al., 2017). Fig. 5 illustrates the comprehensive mechanism of fungal-mediated plastic biodegradation. The process begins when fungi colonize the surface of plastic materials, forming biofilms that facilitate close interaction with the polymer. To break down these otherwise resistant compounds, fungi secrete a variety of extracellular enzymes, most notably hydrolases and oxidoreductases. These enzymes act synergistically to initiate bio-deterioration, weakening the polymer matrix, and promote fragmentation, whereby long polymer chains are cleaved into smaller oligomers and monomers. Once reduced to low-molecular-weight compounds, these products can be assimilated into fungal cells through transport systems and subsequently metabolized via central metabolic pathways. The process ultimately culminates in mineralization, resulting in the conversion of plastic-derived carbon into simpler, non-toxic end-products such as CO₂, H₂O, and fungal biomass (Okal et al., 2023).

Like bacteria, fungi are widely distributed across diverse ecosystems and play a crucial role in the transformation and mobilization of environmental chemicals through the secretion of a broad range of hydrolytic enzymes. Their strong biodegradation potential is largely attributed to a versatile enzymatic repertoire capable of catalyzing various reactions (Olicón-Hernández et al., 2017). Fungal intracellular enzymes, particularly those in the cytochrome P_450_ family, contribute to adaptation and detoxification processes by facilitating oxidation (e.g., epoxidases) and conjugation (e.g., transferases) reactions (Esteves et al., 2021). Additionally, fungi possess an extracellular enzymatic system that supports the breakdown of complex polymers and is divided into two functional subsystems. The first, a hydrolytic subsystem, produces hydrolases involved in the degradation of polysaccharides. The second is a nonspecific oxidative subsystem, which targets complex and recalcitrant structures through the action of oxidoreductases. This subsystem includes enzymes such as dye-decolorizing peroxidases, laccases, manganese peroxidase, versatile peroxidases, lignin peroxidase, and select peroxygenases (Gupta et al., 2023). These unique enzymatic capabilities position fungi as promising candidates for the biodegradation of MPs, particularly in environments contaminated with persistent polymeric pollutants (Robinson, 2015).

They possess the capability to adhere to and reduce the hydrophobicity of MPs by catalyzing the formation of different chemical bonds, such as ester, carbonyl, and carboxyl functional groups (Lai et al., 2025). Fungi are particularly adept at degrading complex-structured polymers, such as aromatic compounds, making them especially effective for the biodegradation of PVC (Ibrahim et al., 2024). Recent research has delved into fungal-mediated degradation of MPs, highlighting their potential to utilize MPs as a carbon source. Noteworthy genera associated with the degradation processes of diverse polymers, including PE, PP, and PET, encompass *Aspergillus niger, Zalerion maritimum*, and *Penicillium simplicissimum* Temporiti et al., 2022). These organisms play pivotal roles in mitigating the hydrophobic nature of polymers while catalyzing the formation of a diverse array of chemical linkages, including ester, carboxyl, and carbonyl functional groups (de Oliveira et al., 2020; Temporiti et al., 2022). Several fungal strains such as *Penicillium chrysogenum, Solani, Aspergillus tubingensis, Aspergillus fumigatus, Pestalotiopsis microspore Cladosporium*, and *pseudocladosporioides* have exhibited the capability to degrade polyurethane (Okal et al., 2023). Fungal species *Aspergillus tubingensis* and *Aspergillus favus*, isolated from maritime coastal environments, have displayed the ability to degrade HDPE (Okal et al., 2023). Recent investigations have shown promising outcomes with *Mucor circinelloides* and *Aspergillus favus*, both sourced from municipal waste, in the degradation of low-density PE. Treatment of MPs, such as PE, with substances like nitric acid and sodium hydroxide has been found to expedite degradation by *Aspergillus niger* (Ekanayaka et al., 2022). However, employing physical pretreatment methods, such as thermo-oxidization at 80 °C for a duration of fifteen days, is necessary to impede the degradation of low-density PE by *Penicillium pinophilum* and *A. niger* (Ekanayaka et al., 2022; Zeghal et al., 2021; Zhai et al., 2023). Notably, fungi belonging to the *Aspergillus* genus exhibit remarkable efficiency in degrading LDPE, surpassing certain bacteria from genera such as *Brevibacillus, Bacillus, Cellulosimicrobium, Pseudomonas, Ochrobactrum*, and *Lysinibacillus* (Zhang et al., 2020).

Isolates of Aspergillus japonicus and A. niger, recovered from PE-polluted sites, demonstrated notable PE degradation capabilities, resulting in weight losses of approximately 8 % and 12 %, respectively (Alariqi et al., 2006). Zalerion maritimum, a marine fungus isolated from coastal waters, exhibited sustained growth in minimal media containing PE as the sole carbon source, highlighting its potential application in the bioremediation of MPs-contaminated marine environments (de Oliveira et al., 2020; Temporiti et al., 2022). In another study, Pestalotiopsis microspora was investigated for its ability to degrade polyurethane, which led to the identification of a serine hydrolase enzyme involved in PU breakdown—an important advancement in MPs degradation strategies (Okal et al., 2023). Penicillium chrysogenum NS10 and Penicillium oxalicum NS4 were identified by Ojha et al. (2017) as effective degraders of both LDPE and HDPE. Over time, further fungal colonization was observed, including genera such as Aspergillus, Alternaria alternata, Geotrichum candidum, Cladosporium spp., additional Penicillium spp., Ulocladium atrum, and Rhodotorula rubra (Arif et al., 2024). Notably, fungal dominance on the plastic surfaces became more pronounced after approximately 56 weeks of exposure. In their study, Webb et al. (2000) investigated polyvinyl chloride samples containing identical plasticizers through in situ assessments. They observed the earliest and most rapid colonization by Aureobasidium pullulans, occurring within 25 to 40 weeks. This was followed by the colonization of Rhodotorula aurantiaca and Kluyveromyces sp., which appeared after 80 weeks of exposure. However, it is important to note that the study did not report any evidence or measurements related to the actual biodegradation of the PVC materials under investigation (Webb et al., 2000). While the studies mainly documented successful surface colonization, which serves as a pivotal precursor to subsequent biodegradation, they do not definitively establish biodegradation itself. Notably, biodegradation of polypropylene has been reported with *Rhizopus arrhizus* and *Aspergillus niger*, while *Humicola insolens* and *Penicillium* sp. have demonstrated efficacy in degrading polyethylene terephthalate (Ronkvist et al., 2009).

### 5.3 Microalgae

Exploring algae species with robust biodegradation capabilities for polymeric compounds offers a promising avenue for MPs remediation. Microalgae thrive in photoautotrophic conditions and utilize atmospheric CO_2_ as their sole carbon source for growth, thereby eliminating the need for inorganic or organic carbon sources (Cheah et al., 2023). Microalgae demonstrate the ability to degrade polymeric materials through the actions of their toxins and enzymes, showcasing adaptability across various environmental conditions and a minimal requirement for abundant carbon sources (Renuka et al., 2021; Song et al., 2020). Within wastewater streams, they colonize plastic surfaces and produce enzymes that aid in plastic degradation, as well as in the enhancement of proteins and carbohydrates, thereby promoting growth. Recent SEM investigations have revealed that algal-driven colonization processes can induce degradation or fragmentation of polyethylene on sheet surfaces (Lagarde et al., 2016). Notably, algae have demonstrated prolific growth on synthetic substrates like polyethylene surfaces in sewage water, with these colony-forming algae presenting minimal risks and toxicity concerns. The biodegradation process of MPs begins with algae adhering to surfaces through the synthesis of extracellular polysaccharide (Sarmah and Rout, 2018). Evidence suggests that microalgae utilize the polymer as a carbon source, as indicated by the higher cellular content of carbohydrates and proteins observed in species growing on polyethylene surfaces (Cheah et al., 2023; Chia et al., 2020). Previous studies have documented various degradation mechanisms, including corrosion, fouling, penetration, hydrolysis, and diffusion-mediated breakdown of leaching components and pigment coloring within polymers (Kumar et al., 2017; Chettri et al., 2023). The enzymatic biodegradation process, particularly involving PETase, is susceptible to ambient temperature conditions and PET structure. Thermal treatment enhances plastic biodegradability by certain bacteria, amplifying the effects of enzymes on long-chain polymer molecules and ultimately facilitating their cleavage or degradation (Zahid et al., 2024). *Anabaena spiroides* demonstrated significant potential in degrading LDPE, achieving a degradation efficiency of 8.19 %. Additionally, the green alga Scenedesmus dimorphus and the diatom Navicula pupula exhibited LDPE degradation rates of 3.75 % and 4.43 %, respectively. Freshwater cyanobacteria such as Oscillatoria subbrevis and Phormidium lucidum, which are easily isolated and widely distributed, were also found to effectively colonize polyethylene surfaces and induce LDPE biodegradation without the need for pro-oxidant additives or pretreatment procedures (Kumar et al., 2017; Sarmah and Rout, 2018). BPA, a common estrogenic component found in polymers, has been successfully degraded by various bacteria and algae strains, including *Chlorella vulgaris, Stephanodiscus hantzschii*, and *Micrococcus mexicanus* (Fikarová et al., 2019). The growth of biofilms on polymer surfaces is often associated with MPs degradation.

Cyanobacterial species within these biofilms, along with diatoms, play vital roles in photosynthesis. Notably, several cyanobacterial strains from genera such as *Calothrix, Microcystis, Prochlorothrix, Pleurocapsa, Leptolyngbya, Synechococcus, Rivularia*, and *Scytonema* have demonstrated the ability to form biofilms on MPs-based polymers (Amaral-Zettler et al., 2020; Gupta et al., 2023). Moreover, advancements in genetic engineering enable the synthesis and release of essential enzymes required for plastic degradation from mutant microalgal cell factories. For example, the genetic modification of the green microalga *Chlamydomonas reinhardtii* has enabled the production of PET hydrolase, an enzyme with the capability to degrade films consisting of terephthalic acid and polyethylene terephthalate (Perozeni and Baier, 2023). Similarly, effective genetic modifications in *P. tricornutum* have led to the production of PET hydrolase, exhibiting catalytic activity against both polyethylene terephthalate and its copolymers. With their straightforward cultivation and ability to utilize plastic monomers as carbon sources while synthesizing plastic-degrading enzymes, microalgae have emerged as promising candidates for MPs degradation (Nabi et al., 2023). Advancements in genetic manipulation have paved the way for enhancing the degrading capabilities of algal strains, offering a potentially environmentally beneficial approach for biologically decomposing polyethylene terephthalate.

## 6 Enhancement of biodegradation of MPs

**Fig. 6:**
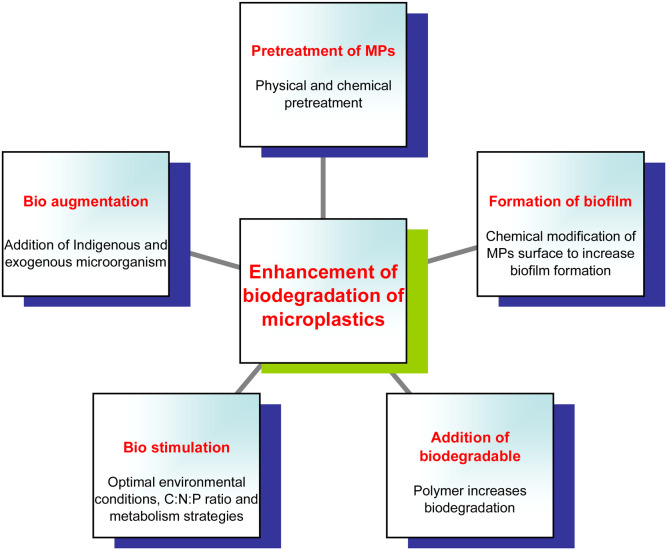
Methods to enhance MPs biodegradation.

The biodegradation of polymers such as PE, PP, PS, and PVC poses significant challenges due to their lack of hydrolyzable functional groups. Enhancing MPs biodegradation can be achieved by optimizing biological and environmental factors that influence microbial activity and polymer breakdown. Pretreatment of plastics through physical or chemical methods improves surface properties, facilitating microbial colonization and enzyme access (Mohanan et al., 2020). The initial breakdown and subsequent reduction in the molecular weight of these MPs in the environment are largely due to the synergistic action of abiotic and biotic factors (Wei and Zimmermann, 2017). Genetic engineering and microbial consortia further boost degradation efficiency, while optimizing environmental conditions enhances enzymatic performance. The use of nanomaterials or biosurfactants also strengthens polymer–microbe interactions, collectively advancing sustainable plastic waste biodegradation (Mohanan et al., 2020). Various strategies have been investigated to enhance the biodegradation of MPs, as illustrated in Fig. 6. Physical and chemical pretreatments are generally employed to disrupt the chemical bonds within the MPs structure, thereby facilitating easier biodegradation. Common chemical agents for surface pretreatment include nitric acid, which oxidizes the polymer and introduces double bonds and carbonyl groups into the polymer backbone (Artham and Doble, 2008). Ozone is also utilized to accelerate the aging of MPs. Physical pretreatment methods encompass irradiation and thermal treatment. UV-irradiation is commonly employed to initiate photooxidative degradation of plastics, as its wavelengths promote the formation of free radicals and the subsequent cleavage of polymer chains (Amanna and Rakshit, 2025). In a notable study by Roy et al. (2008), a bacterial consortium comprising Bacillus pumilus, Bacillus cereus, and Bacillus halodenitrificans was utilized to biodegrade LDPE films that contained trace amounts of cobalt stearate as a prooxidant. The UV-induced oxidative pretreatment facilitated microbial colonization and enhanced the overall degradation efficiency of the polymer.

Prior to analysis, the samples were subjected to UV-B irradiation within the range of 280–315 nm. Following a two-week period, a substantial improvement in biodegradation was observed: untreated samples experienced a mere 0.25 % weight loss, while pretreated samples exhibited a notable 8.40 % reduction (Zhang et al., 2025). Vimala and Mathew (2016) demonstrated that UV pretreatment of PE, combined with the application of a biosurfactant, enhanced microbial degradation, resulting in a 9.26 % reduction in polymer weight after 30 days. Similarly, Esmaeili et al. (2013) employed a mixed microbial consortium consisting of Lysinibacillus xylanilyticus and Aspergillus niger to degrade LDPE films. Over a 126-day incubation period, the untreated LDPE samples exhibited a 15.8 % degradation, while UV-pretreated samples showed a markedly higher degradation rate of 29.5 %, underscoring the efficacy of photooxidative pretreatment in enhancing biodegradation.

Biological processes are intricately specific, relying on the enzymatic activity of microbial communities, optimal conditions for the growth and reproduction of microorganisms, and a balanced nutrient and substrate ratio. Thus, to enhance bioremediation efficiency, techniques like bioaugmentation and bio stimulation are employed. Bio stimulation entails creating conditions conducive to microbial degradation of substrates, often necessitating the external supplementation of deficient nutrients (Tyagi et al., 2011). Each microorganism thrives within a specific C:N:P ratio, and adjusting this balance can greatly enhance biodegradation. Incorporating additives such as glucose, K_2_HPO_4_, KNO_3_, and NaNO_3_ can create these favorable conditions (Chen and Chen, 2021). Additionally, microorganisms are sensitive to environmental variables like acidity fluctuations, which can significantly influence their activity levels (Joutey et al., 2013). Microbial activity relies heavily on maintaining optimal pH levels, which vary depending on the microorganism; bacteria thrive in neutral pH, whereas fungi prefer acidic environments (Martinez-Toledo et al., 2018). Adequate oxygen availability is essential for maintaining the metabolic activity of aerobic microorganisms, while optimal temperature ranges vary across microbial species. Together, these environmental factors significantly influence microbial growth, spatial distribution, and the overall efficiency of biodegradation processes (Gonzalez and Aranda, 2023). Several microbial genera are known for their ability to produce biosurfactants, which enhance the bioavailability of hydrophobic pollutants and facilitate degradation. These include Acinetobacter, Arthrobacter, Bacillus, Halomonas, Enterobacter, Rhodococcus, and Pseudomonas (Kuyukina et al., 2005).

Typically, microorganisms produce enzymes to break down primary substrates, their primary energy sources. However, these enzymes may also degrade secondary substrates like MPs polymers, although these often fail to effectively stimulate enzyme production. To enhance biostimulation, co-metabolism strategies are employed, wherein primary substrates are introduced into contaminated environments to stimulate indigenous microorganisms to produce specific degradation enzymes (Goswami et al., 2018). This approach boosts microbial activity without the primary substrate itself being the target of degradation. Indigenous microbial communities, naturally present in polluted habitats, play a pivotal role in biodegradation processes due to their adaptation to local environmental conditions, making them especially effective in site-specific bioremediation efforts (Semrany et al., 2012). Ibiene et al. (2013) successfully isolated Bacillus subtilis and Bacillus mycoides from mangrove soil in the Niger Delta and demonstrated their effectiveness in degrading both low-density and high-density polyethylene. In a related study, Auta et al. (2017) isolated Bacillus gottheilii and B. cereus from the same environment and utilized them to degrade a range of plastic polymers, including PE, PP, PS, and PET microparticles. These findings underscore the potential of indigenous microorganisms in plastic biodegradation. Moreover, increasing the concentration of native microbial populations has been shown to significantly improve degradation efficiency (Matthies et al., 1997).

In cases where indigenous microbial communities lack the capacity to degrade pollutants effectively, the introduction of exogenous microorganisms known as bioaugmentation may be employed (Tao et al., 2018). However, this strategy presents several challenges. Exogenous strains must adapt to unfamiliar and often harsh environmental conditions, which may necessitate modification of local parameters such as pH, temperature, and nutrient availability to ensure successful colonization. Prior laboratory cultivation is essential for optimizing the growth conditions and maximizing degradation efficiency within a limited timeframe. Nonetheless, the transition from controlled laboratory settings to complex, variable field conditions can lead to reduced viability or even extinction of introduced species. Furthermore, exogenous microorganisms may outcompete native species due to traits such as rapid growth, high fecundity, and metabolic versatility (Haque and Gazi-Khan, 2025). This competition may disrupt the structure, diversity, and ecological function of the indigenous microbial community, raising concerns regarding long-term ecological stability.

## 7 Future prospects and challenges

Although considerable progress has been made in elucidating microbial contributions to MPs degradation, multiple barriers constrain their translation into effective large-scale remediation strategies. Current microbial degradation kinetics remain markedly slow under environmentally relevant conditions, where fluctuating parameters such as temperature, salinity, pH, and co-contaminant load significantly modulate microbial activity, enzyme stability, and substrate accessibility. Moreover, incomplete mineralization frequently leads to the accumulation of micro- and nanofragments or intermediate metabolites, many of which retain ecotoxicological properties. To date, only a narrow spectrum of bacterial and fungal taxa most notably Ideonella sakaiensis, Pseudomonas spp., Bacillus spp., Aspergillus, and Fusarium have demonstrated measurable plastic-degrading capacity, and their performance is typically confined to controlled laboratory conditions. The limited diversity of characterized strains and enzymes with broad substrate specificity represents a fundamental bottleneck, further restricting the ecological applicability of these pathways.

Addressing these limitations necessitates a multipronged research agenda. Protein engineering and synthetic biology should be leveraged to improve the catalytic efficiency, thermostability, and polymer selectivity of key hydrolases and oxidoreductases, including PETase, MHETase, cutinases, and laccases. Omics-driven investigations (genomics, transcriptomics, proteomics, and metabolomics) offer a powerful platform to uncover novel catabolic pathways and enzyme systems from plastisphere-associated microbiomes, marine ecosystems, and extremophiles. The rational design of synthetic microbial consortia, integrating complementary metabolic functions, may provide synergistic capacity for complete mineralization. In parallel, hybrid remediation frameworks that integrate physicochemical pretreatments such as photothermal oxidation, catalytic activation, or plasma processing with microbial or enzymatic systems hold promise for substantially accelerating degradation rates. Future efforts must also prioritize systematic ecotoxicological evaluation of degradation intermediates and pilot-scale, in situ trials to assess scalability, resilience, and long-term environmental safety. Ultimately, realizing the potential of microbial pathways for MPs degradation will require convergence of biotechnology, systems biology, and environmental engineering, alongside robust ecological validation, to develop scalable, sustainable, and safe solutions for mitigating global plastic pollution.

## 8 Conclusions

Microplastic pollution constitutes a significant and escalating environmental and ecological challenge, and microbial biodegradation represents a promising, sustainable strategy for its mitigation. Among microorganisms, bacterial taxa such as *Bacillus, Pseudomonas*, and *Ideonella sakaiensis* have demonstrated substantial potential due to their secretion of specific plastic-degrading enzymes, including PETase and MHETase. Fungal species, particularly *Aspergillus* and *Fusarium*, exhibit pronounced depolymerization capabilities via extracellular enzymatic activity, whereas microalgae such as *Chlamydomonas reinhardtii* and *Chlorella vulgaris* offer additional eco-compatible biodegradation pathways. Emerging evidence also highlights insect-associated gut microbiota (e.g., waxworms, mealworms) as novel bioremediation agents capable of initiating plastic depolymerization.

Despite these advancements, microbial degradation rates remain low, and efficiency is strongly governed by critical factors including polymer type and physicochemical properties, microbial community composition, enzymatic activity, and environmental parameters such as temperature, pH, salinity, and the presence of co-contaminants. While laboratory-scale investigations demonstrate feasibility, scaling these processes to field conditions presents substantial challenges, particularly in maintaining microbial viability, enzymatic stability, and effective polymer contact under heterogeneous environmental settings.

Future research should prioritize biotechnological enhancement of microbial and enzymatic performance, including protein engineering, directed evolution, and the design of synthetic microbial consortia to achieve complete polymer mineralization. Integrating microbial approaches with cost-effective bio-based plastic production and process optimization will be critical for sustainable, large-scale application. Additionally, in-depth characterization of microbial ecology, enzymatic pathways, and environmental interactions is necessary to inform the development of robust, scalable, and ecologically safe biodegradation strategies.

In conclusion, bacteria and fungi currently represent the most feasible and effective microbial candidates for microplastic biodegradation. However, the successful translation of these biological strategies into practical environmental solutions will require a synergistic integration of advanced biotechnology, environmental optimization, and systems-level understanding of microbial-plastic interactions. The development of scalable, cost-effective methods for producing bio-based plastics and enhancing the capabilities of microbial strains will be crucial for the widespread application of this approach in mitigating MPs pollution.

## Declaration of competing interest

The authors declare that no conflict of interest in the present manuscript
